# Khat chewing among Ethiopian University Students- a growing concern

**DOI:** 10.1186/1471-2458-14-1198

**Published:** 2014-11-21

**Authors:** Ewenat Gebrehanna, Yemane Berhane, Alemayehu Worku

**Affiliations:** Addis Continental Institute of Public Health, Addis Ababa, Ethiopia; School of Public Health, Addis Ababa University, Addis Ababa, Ethiopia

**Keywords:** Khat chewing, Ethiopia, University students

## Abstract

**Background:**

Khat has amphetamine like effect. Students chew khat to stay alert. It has various negative physical, mental, social and cognitive effects. Poor academic performance has been associated with khat. The purpose of this study was to determine the prevalence and identify factors associated with khat chewing among Ethiopian University students.

**Methods:**

A cross sectional study was conducted on Bahir Dar University Students. A self-administered questionnaire was completed by 3268 students. Proportion was calculated to estimate prevalence of khat chewing. Logistic regression was used to identify factors associated with khat chewing.

**Results:**

Lifetime prevalence of khat chewing was 24% (95% Confidence Interval: 22.5%, 26.6%). Half of these are current khat users with a prevalence of 12.7% (95% Confidence Interval: 11.5%, 13.9%). Male students Adjusted Odds Ratio (AOR) = 3.3 (95% Confidence Interval: 1.8, 6.0), students living in off campus housing AOR = 3.0 (95% Confidence Interval: 1.5, 6.0), students who have khat user friends AOR = 4.2 (95% Confidence Interval: 2.6, 6.9), and students who perceive khat use improves academic performance AOR = 6.6 (95% Confidence Interval: 4.6, 9.5) are more likely to use khat.

**Conclusions:**

Prevalence of current use of khat reported in this study is higher than recent study done on university students in Ethiopia and heavily influenced with peer practice.

## Background

Khat is a plant with amphetamine like characteristics with effects including insomnia, euphoria, decreased fatigue and suppressed appetite when chewed [1]. Frequent and chronic khat use has been associated with various consequences. These include substance dependency [2], early sexual debut [3] unprotected sex [4] mental health issues [5, 6] and with various social, cognitive and financial problems [7–10].

Ethiopia is one of several countries in Africa and the Middle East where khat chewing is common. A national study reported 23.0% of out-of-school and 7.5% of in-school adolescents use khat [4]. A separate study estimated prevalence of khat use in the general population of Ethiopia was 27.3% among men and 11.0% among women of 15–49 years in 2011 [11].

Khat is consumed by students when they wish to study for long hours especially during examination periods [12]. In Ethiopia there have been few studies of khat use among university students. The available studies were either done before the expansion of public universities and increased admission loads or represent only a single faculty/department of a university which might affect estimation of khat use among university students. The study conducted in 2002 among Gondar School of Medical Science and Bahir Dar polytechnic - and Pedagogy Colleges reveled a prevalence of 17.5% [13]. This study was conducted before universities expanded to the current level as explained later on. Other, more recent studies, reported prevalence of 14.4% from a study done on technology and pharmacy faculties of Addis Ababa University [14]; 2.3% among Medical faculty students of Addis Ababa University [15]; 6.3% from Debremarkos polytechnic college [16]; 27.9% from Axum University [17]; and 12.9% from private and public colleges in Bahir Dar city [18].

Socio-demographic characteristics such as being male, having pee r influence and student seniority have been associated with khat chewing [19–21]. The most common reasons g ven for chewing khat include: staying alert and achieving better concentration at work; increasing motivation when there is a need to work long hours; socializing; and other recreational purposes [13, 22].

Ethiopia has markedly expanded the number and admission capacity of public universities in the last decades. The numbers of public universities under the Federal Ministry of Education (MoE) have increased from less than ten in 2004/2005 academic year to thirty-one in 2013/2014. Currently four additional universities are in line to join these universities [23]. This expansion has also changed the diversity of students. Different regions and cultural groups are better represented. Undergraduate students are assigned to public universities from all over the country by the Ministry of Education of Ethiopia (MoE). Due to this process students at public universities in Ethiopia are composed of different ethnic and cultural backgrounds.

Knowing the prevalence and reasons for khat chewing among university students is important because of its serious health, social and economic consequences. Khat use among university students in Ethiopia has been studied as early as 1988 [24]. However recently the diversity of university students have changed following expansion of higher education in Ethiopia. In addition most recent studies on khat use among Ethiopian university students were done on single faculties or departments, which may not represent the whole university student community. Those factors outside of the university campus, such as living arrangements, that may affect students’ khat use are not well documented. Therefore, this study was designed to assess the prevalence and reasons for khat chewing among university students in the current context of university setups using representative sample size from Bahir Dar University.

## Methods

### Study area

A cross sectional study was conducted among University students of Bahir Dar University, Ethiopia, in June 2012. During the 2011/2012 academic year, Bahir Dar University enrolled 15,066 regular undergraduate students. Of which 32,66 (21.7%) were female [25]. Bahir Dar University has academic units which include 3 faculties, 4 Colleges, 3 Institutes, and two academies.

### Sample size and sampling

Sample size for the study was determined by using a single population formula to determine prevalence and a two population formula to identify factors across year of study. The assumption for sample size calculation was that khat chewing will be affected by the length of stay at university (Year of study). We have calculated sample size for each year of study using Epiinfo version 3.5.1. The maximum sample size was taken for the data collection. With the exception of one academy which didn’t have a regular undergraduate education, all academic units within the university were included. All regular undergraduate students were eligible for the study. The sample size calculated was distributed for each academic unit based on the number of students. Sampling was further refined by taking into consideration the male to female student ratio. Then, students were selected using a simple random sampling procedure from the registrar office lists.

### Study tool

Data were collected using a structured pre-tested, self-administered questionnaire. The questionnaire was developed based on literature and previously-used questionnaires. In addition, a formative qualitative assessment was done in Gondar University, a public university located 180 Km north from Bahir Dar University. Gondar University, also a public university, has student admission, accommodation, and academic systems similar to Bahir Dar University. The surrounding communities both around Gondar and Bahir Dar Universities share similar language and culture. The formative assessment identified current issues around khat use among university students. Some of the issues that arose during the formative assessment include students’ khat chewing habit off campus and how khat chewer students cluster together. Following the formative assessment, a questionnaire was developed, and a pretest was conducted at Gondar University. The questionnaire was prepared and administered in English and Amharic (Ethiopian official language) so that participants could choose either language.

### Data collection

Only students selected for the study were asked to remain in their classroom to complete a self-administered questionnaire. Students were given detailed explanation of the study and how to fill the questionnaire by trained facilitators. As khat use has some level of stigma, a social desirability bias was expected. To minimize this potential bias, students were given the maximum privacy by not allowing instructors or any other member of the Bahir Dar University staff not to enter in the classrooms during data collection and by arranging the seats with enough space between students.

### Measuring prevalence

Khat use was measured as ever and current use. Ever use was defined as khat use experience at any time during their lifetime. Current use was defined as use of khat during the current academic year and classified into three categories: current academic year (September 2011-June 2012); during the current second semester (Feb-June 2012); and during the last four weeks preceding the survey (which was June 2012).

### Habitual use

Current users were classified into habitual and occasional users. Habitual use was defined as khat chewing at least once a week while occasional use was defined as khat chewing less than once in a week.

### Statistical analysis

Prevalence was calculated with 95% confidence interval (CI) to describe ever and current khat use as defined above. Bivariate and multivariable logistic regressions were done to assess association between khat chewing and selected factors. Socio-demographic and economic variables, friends’ characteristics, perceptions about khat, living arrangements, and family history of khat use were considered in the bivariate logistic regression. Only variables associated with khat chewing at p-value 0.2 or less during the bivariate logistic regression were entered into the multivariable logistic regression model. Variables with very low cell values (less than 5) were excluded from logistic regression to maintain the stability of the models.

### Ethical issues

The study protocol was approved by the Ethical Review Board of Bahir Dar University. Informed consent was obtained from each participant and participation into the study was fully voluntary. Study participant students were offered refreshment worth 5 Ethiopian Birr (0.28 USD) at the university student café to compensate the time spent on responding to the survey.

## Results

**Figure 1:**
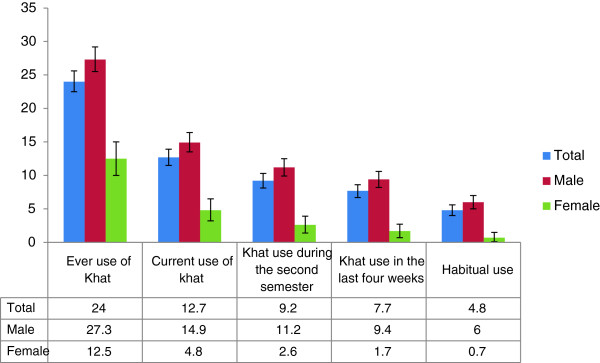
**Prevalence of khat chewing across different reference time by sex among Bahir Dar University Students, June 2012.**

A total of 3,872 students were invited to participate in the study and of which 3,268 completed and returned the self-administered questionnaire. Of those, 3,001 questionnaires were completely filled and included for analysis in this paper. Thus, the overall response rate was 77.5%. The majority of respondent were male (77.6%), Orthodox Christians (81.8%), living on campus (96.1%) (Table 1).The overall prevalence of lifetime khat use is 24.0% (95% CI: 22.5%, 25.6%). Current use of khat is reported by 12.7% (95% CI: 11.5%-13.9%) of students. The prevalence of habitual khat use is reported by 4.8% (95% CI: 4.0%, 5.6%). Khat chewing occurs more frequently among male students than females in all categories (Figure 1).

**Table 1:** **Profile of study participant Bahir Dar University Students, Ethiopia 2012**

	Frequency	Percentage
**Sex**		
Male	2328	77.6
Female	673	22.4
**Year of study**		
Year 1	834	27.8
Year 2	823	27.4
Year 3	824	27.5
Year 4 and above	520	17.3
**Average monthly pocket money**		
Doesn’t receive pocket money	251	8.0
Less than 100 Birr	533	16.9
100-199	646	20.5
200-299	587	18.7
300-399	414	13.2
400-499	238	7.6
500 or above	476	15.1
**Religion**		
Orthodox	2437	81.8
Catholic	19	0.6
Protestant	262	8.8
Muslim	227	7.6
No religion	14	0.5
Others	21	0.7
**On campus housing**		
Yes	2771	96.1
No	114	3.9

An Adjusted Odds Ratio (AOR) was calculated to various factors that are likely to be associated with ever, current (chewing in the current academic year) and habitual khat use (Table 2). Male students were more likely to chew khat in the current academic year as compared to female students [AOR = 3.3 (95% CI: 1.8, 6.0)]. Students who perceive that khat helps to study better were more than 6 times more likely to chew khat in the current academic year as compared to those who do not [AOR = 6.6 (95% CI: 4.6, 9.5)]. Students who live in off campus housing were 3 times more likely to chew khat in the current academic year as compared to those students who reside on campus [AOR = 3.0 (95% CI: 1.5, 6.0)]. Students were more likely to chew khat if they have close friends who are khat chewers compared to those who do not have friends who chew khat at all [AOR = 4.2 (95% CI: 2.6, 6.9)]. IStudents that reported family history of khat chewing were more likely to chew khat than students who reported no family history of khat use [AOR = 2.8 (1.9, 4.2)]. These factors were found to be cross-cutting factors across the three categories of khat use (Table 2).

**Table 2:** **Factors associated with ever, current and habitual use of khat among Bahir Dar University students, Ethiopia 2012**

	Ever use of khat		Khat use (current academic year)		Habitual use of khat	
	COR (95% CI)	AOR (95% CI)	COR (95% CI)	AOR (95% CI)	COR (95% CI)	AOR (95% CI)
**Sex**						
Female	1.0	1.0	1.0	1.0	1.0	1.0
Male	2.6(2.0,3.4)	*3.1(2.0, 4.8)* ^***^	3.5(2.4, 5.1)	*3.3(1.8, 6.0)* ^*^	8.1(3.3, 20.0)	*4.5(1.4, 14.4)* ^*^
Year of Study						
Year 1	1.0	1.0	1.0	1.0	1.0	1.0
Year 2	1.4(1.1,1.8)	*1.5(1.1, 2.2)* ^***^	1.3(0.9, 1.7)	0.9(0.6, 1.6)	1.5(0.8, 2.7)	0.8(0.3, 1.8)
Year 3	1.2(0.9, 1.5)	*1.7(1.1, 2.5)* ^*^	1.1(0.8, 1.5)	1.3(0.8, 2.2)	1.4(0.8, 2.4)	1.2(0.5, 2.8)
Year 4 and above	2.0(1.6, 2.6)	1.3(0.8, 1.9)	2.4(1.7, 3.2)	1.1(0.7, 1.8)	4.5(2.7, 7.4)	2.0(1.0, 4.2)
**Religion**						
Orthodox Christian	1.0	1.0	1.0	1.0	1.0	1.0
Catholic	2.7(1.0-7.0)	0.9(0.2, 3.9)	3.6(1.3, 10.3)	0.9(0.2, 5.5)	3.3(0.7, 14.6)	0.26(0.01, 4.35)
Protestant	1.1(0.8, 1.5)	1.0(0.6, 1.6)	1.1(0.7, 1.7)	0.9(0.5, 1.6)	1.0(0.5, 2.0)	1.0(0.4, 2.6)
Muslim	4.3(3.3, 5.7)	*1.7(1.1, 2.7)* ^*^	4.0(3.0, 5.5)	*2.1(1.2, 3.5)* ^**^	3.4(2.2, 5.4)	1.5(0.7, 3.0)
No religion	6.1(2.0, 18.7)	0.8(0.1, 5.9)	13.9(4.5, 42.8)	2.3(0.3, 16.2)	7.4(2.0, 27.3)	1.0(0.2, 5.3)
Other	1.9(0.8, 4.7)	1.3(0.3, 5.1)	3.5(1.3, 9.0)	2.5(0.6, 10.7)	4.3(1.3, 15.1)	1.7(0.3, 11.3)
**Childhood residence**						
Rural	1.0	1.0	1.0	1.0	1.0	1.0
Small towns	2.7(2.2, 3.4)	*1.8(1.3, 2.6)* ^**^	3.3(2.4, 4.4)	1.6(1.0, 2.6)	5.7(3.2, 10.3)	1.4(1.0, 5.5)
Big cities	3.3(2.6, 4.2)	*2.5(1.7, 3.8)* ^***^	4.7(3.4, 6.4)	*2.2(1.3, 3.7)* ^**^	7.7(4.3, 13.9)	1.2(0.5, 3.2)
**Average monthly pocket money received from families and friends**						
Do not get money	1.0	1.0	1.0	1.0	1.0	1.0
< 100 Birr	0.4(0.3, 0.7)	0.9(0.5, 1.7)	0.4 (0.2, 0.8)	1.0(0.4, 2.5)	0.3(0.1, 0.8)	0.9(0.2, 3.7)
100-199 Birr	0.8(0.5, 1.1)	1.1(0.6, 2.0)	0.7(0.4, 1.2)	1.0(0.4, 2.0)	0.4(0.1,0.9)	0.3(0.1, 1.1)
200-299 Birr	1.1(0.8, 1.6)	1.4(0.8, 2.5)	1.2(0.7, 2.0)	1.3(0.6, 2.8)	1.0(0.4, 2.2)	0.9(0.3, 2.6)
300-399 Birr	1.5(1.0, 2.1)	1.5(0.8, 2.7)	2.1(1.2, 3.4)	2.1(1.0, 4.3)	1.3(0.6, 3.0)	0.9(0.3, 2.6)
400-499 Birr	1.4(0.9, 2.2)	1.0(0.5, 2.0)	2.5(1.5, 4.3)	2.2(1.0, 4.9)	3.3(1.5, 7.2)	2.9(1.0, 8.5)
500 and above	1.7(1.2, 2.4)	1.2(0.6, 2.2)	2.8(1.7, 4.6)	1.8(0.9, 3.8)	2.7(1.3, 5.7)	0.8(0.3, 2.3)
**Off Campus Housing**						
No	1.0	1.0	1.0	1.0	1.0	1.0
Yes	5.0(3.4, 7.4)	*2.1(1.1, 4.1)* ^*^	7.4(5.0, 11.0)	*3.0(1.5, 6.0)* ^**^	12.4(7.9, 19.7)	*6.3(2.9, 13.8)* ^***^
**Family History of Khat chewing**						
No	1.0	1.0	1.0	1.0	1.0	1.0
Yes	6.9(5.6, 8.5)	*3.9(2.8, 5.5)* ^***^	6.8(5.3, 8.6)	*2.8(1.9, 4.2)* ^***^	6.6(4.6, 9.3)	*2.4(1.3, 4.1)* ^**^
**Close friends chew Khat**						
No	1.0	1.0	1.0	1.0	1.0	1.0
Yes	6.6(5.3, 8.3)	*2.6(1.9, 3.7)* ^***^	13.4(9.3, 19.3)	*4.2(2.6, 6.9*)^***^	18.6(9.0, 38.1)	*3.6(1.4, 9.3)* ^**^
**Dorm mates chew khat**						
No	1.0	1.0	1.0	1.0	1.0	1.0
Yes	3.6(2.9, 4.3)	*1.4(1.1, 2.0)* ^*^	5.5(4.3, 7.2)	*1.9(1.3, 2.9)* ^**^	7.8(5.0, 12.4)	1.8(0.9, 3.4)
**Believe that Khat helps to study better**						
No	1.0	1.0	1.0	1.0	1.0	1.0
Yes	9.7(7.7, 12.3)	*7.5(5.3, 10.4)* ^***^	12.6(9.7, 16.3)	*6.6(4.6, 9.5)* ^***^	20.2(13.3, 30.6)	*8.8(5.0,15.4)* ^***^

*- P < 0.05 **- p < 0.01 ***- p < 0.001.

## Discussion

About a fourth of university students, 24.0%, have ever chewed khat, and about 1 in 8 reported chewing khat during the academic year in which the study was conducted. Male students were more likely to chew khat. Students living in off campus housing, those having a chewer friend, and those having a positive perception of khat benefits for studying well were also more likely to use khat.

This study provides a more accurate prevalence of khat use as compared to most of the recent studies on khat use among university students in Ethiopia. This study has much larger sample size which represent all the undergraduate students compared to previous studies conducted among University students in Ethiopia [13, 15]. In addition, male and female students were selected for the study proportionally considering the actual male to female student ratio at Bahir Dar University during the study period thus avoiding representation bias.

Majority of the non-response rate in this study was accounted for by the absence of the students in the graduating class who left the campus for internships off campus during the study period. The absence of the students is likely to underestimate the prevalence of khat use. Due to this non-response, we might not have detected the actual difference of khat use across study years. Previous study on university students of Ethiopia has shown an increase combined prevalence of khat use and cigarette smoking [13]. Studies elsewhere have shown increased prevalence of substance use among college students as year of study increases [26].

The current study has reported a 24.0% lifetime use of khat which is similar to previously reported lifetime prevalence in 2002 [13]. However, recent use of khat measured during the four-week data collection period was different from 17.5% in 2002 [13] to 7.7% in this study. Another study done among Addis Ababa University Medical students reported four week khat use prevalence of 3.7%, which is lower than the 2002 report [15] as well as the current study. Current reports of lower khat use among university students may not be a real decline but due to change in social and demographic diversity of students that followed the wide expansion of higher education in Ethiopia after 2002 [27]. Another explanation is the various definitions used to define current use may have caused the difference. Some of the studies done on khat use among university students didn’t establish clear definitions of current khat use [14, 15, 18].

In this study, prevalence of khat chewingwas higher among male students when measured across different time periods. This is consistent with several studies conducted among higher learning institutions in Ethiopia and elsewhere [13, 15, 20, 21, 28]. Substance use is mediated by gender identities. Patriarchal societies accept substance use among men more easily than substance use among women allowing men to use substances more than women [29, 30]. Men have also more opportunities for substance use putting them at higher risk [26].

This study showed that students who have close friends who chew khat are more likely to use khat. Previous evidences have similar association with khat chewing and friends’ khat use [13, 15]. Khat chewing usually starts during adolescence period and is introduced by friends and peers [31, 32]. Other substance use studies have documented that the closeness of friendship as well as the level of friends’ influence are strong indicators of substance use among young people [33, 34].

Family history of khat use is associated with current khat use by students in this study. This is consistent with previous studies which reported association between family history and khat use [13, 15, 35]. This could be due to acceptance of khat use as normative behavior by students whose family members use khat.

Students who reside in off campus accommodation are more likely to be current and habitual chewers. This could be as a result of students’ choice of living with other students who have similar interests together exposing them to peer influence [33]. Off campus accommodation is a choice made by individual students. Students with particular characteristics and behavior may choose to live together. This arrangement could be a proximal indicator of students’ higher risk of khat and other substance use [36].

Expectation of khat to help students study was the strongest predictor for khat use in this study. The main reason given for chewing khat by students is to concentrate and study well [13, 15]. This is similar to other substance use studies among university students where expectation of substance to enhance academic performance have been associated with use [37–39]. Studies that examine the association between khat use cognitive response and learning had reported an opposing finding to the positive expectation [10]. Khat has been linked with diminished cognitive capacity and poor academic performance. Khat chewing university students in Ethiopia were found to have a lower mean Cumulative Grade Point Average (CGPA) as compared to non-chewer students [40]. Poorer academic performance was also documented in students in Saudi Arabia [41]. While there has been no evidence that khat affects already stored knowledge, there is evidence that shows its interference in active learning while using it [10]. A study on methamphetamine, use has shown similar relationship between learning and use, where learning is affected once an individual starts using the substance [42]. Khat has similar effects with amphetamine [43, 44], which is in turn has weaker, but similar, effect to methamphetamine [45].

## Conclusion

This study has shown that khat use is closely associated with students’ contextual environment and expectation of khat. The finding that students have positive expectation of khat for their academic performance conflicts with existing evidences of negative effects of khat on academic performance. This study has also identified that khat use among university students is highly influenced by peer and family khat use.
